# Intracellular survival of *Streptococcus pneumoniae* in human alveolar macrophages is augmented with HIV infection

**DOI:** 10.3389/fimmu.2022.992659

**Published:** 2022-09-20

**Authors:** Tinashe K. Nyazika, Lusako Sibale, Joseph Phiri, Megan De Ste Croix, Zydrune Jasiunaite, Christopher Mkandawire, Rose Malamba, Anstead Kankwatira, Miriam Manduwa, Daniela M. Ferreira, Tonney S. Nyirenda, Marco R. Oggioni, Henry C. Mwandumba, Kondwani C. Jambo

**Affiliations:** ^1^ Malawi-Liverpool-Wellcome Trust Clinical Research Programme, University of Malawi College of Medicine, Blantyre, Malawi; ^2^ Department of Clinical Sciences, Liverpool School of Tropical Medicine, Liverpool, United Kingdom; ^3^ Department of Genetics and Genome Biology, University of Leicester, Leicester, United Kingdom; ^4^ Department of Pathology, College of Medicine, University of Malawi, Blantyre, Malawi; ^5^ Dipartimento di Farmacia e Biotecnologie, Universita di Bologna, Bologna, Italy

**Keywords:** *Streptococcus pneumoniae*, alveolar macrophages, HIV, intracellular killing, lung

## Abstract

People Living with HIV (PLHIV) are at an increased risk of pneumococcal pneumonia than HIV-uninfected adults, but the reasons for this are still not well understood. We investigated whether alveolar macrophages (AM) mediated control of pneumococcal infection is impaired in PLHIV compared to HIV-uninfected adults. We assessed anti-bactericidal activity against *Streptococcus pneumoniae* of primary human AM obtained from PLHIV and HIV-uninfected adults. We found that pneumococcus survived intracellularly in AMs at least 24 hours post *ex vivo* infection, and this was more frequent in PLHIV than HIV-uninfected adults. Corroborating these findings, *in vivo* evidence showed that PLHIV had a higher propensity for harboring *S. pneumoniae* within their AMs than HIV-uninfected adults. Moreover, bacterial intracellular survival in AMs was associated with extracellular propagation of pneumococcal infection. Our data suggest that failure of AMs to eliminate *S. pneumoniae* intracellularly could contribute to the increased risk of pneumococcal pneumonia in PLHIV.

## Introduction

*Streptococcus pneumoniae*, the pneumococcus, is the leading bacterial cause of community-acquired pneumonia in children under 5-years of age, the elderly and people living with HIV (PLHIV) (1–3). The pneumococcus frequently colonizes the nasopharynx, especially in under-fives and PLHIV (4–7), and this is widely-accepted as a prerequisite for disease (8, 9). Globally, pneumococcus serotype 3 (ST3) is of major concern, since effectiveness of the pneumococcal conjugate vaccines (PCV) against it remain uncertain. Specifically, there is limited reduction in ST3 disease compared to the other vaccine serotypes in the era of PCV (7, 10, 11).

Recent evidence in humans suggests that the pneumococcus migrates into the lung through micro-aspiration, where if not successfully cleared leads to pneumococcal pneumonia (12, 13). Airway phagocytic cells, including alveolar macrophages (AM) and neutrophils, control extracellular bacteria including the pneumococcus, through bacterial internalization, phagolysosomal killing, release of microbiocidal factors, and metal intoxication (14–17). Internalization of pneumococci by phagocytes is enhanced by complement and antibody opsonization (18–20). However, the pneumococcus can evade intracellular killing and survive in macrophages (12, 14). In murine and porcine models, pneumococci have been shown to replicate within CD169^+^ metalophillic splenic macrophages, from where they further propagate infection (14). Furthermore, in the experimental human challenge model, between 29 and 49 days post challenge, 41% of the colonized volunteers had detectable serotype 6B DNA in their BAL fluid, and with some harboring intracellular serotype 6B pneumococci in their AMs (12). While, none of the non-colonized individuals had evidence of persistent serotype 6B in the lung. Together, these findings indicate a potential underappreciated role of AM in the pathogenesis of pneumococcal disease.

However, it is still unclear whether *S. pneumoniae* has increased propensity to evade intracellular killing in AM from PLHIV, as this could contribute to their increased susceptibility to pneumococcal pneumonia. Using *ex vivo* and *in vivo* infection, we show that AM from PLHIV exhibit poor intracellular killing of pneumococci and demonstrated a role of intracellular survival in continued propagation of infection. These data suggest that AM from PLHIV are less able to fully clear pneumococcal infection, and this could contribute to the increased proclivity for pneumococcal pneumonia in PLHIV.

## Methods

### Study design and participants

In a cross-sectional study, we recruited asymptomatic adults >18 years of age comprising of three groups namely, HIV-uninfected, PLHIV on short-term (<3 months) antiretroviral therapy (ART) and long-term (>3 years) ART. All PLHIV were on tenofovir, lamivudine and Efavirenz ART regimen according to national guidelines. Participants were recruited from Queen Elizabeth Central Hospital Voluntary Counselling and Testing (VCT) clinic in Blantyre, Malawi. Participants were excluded during screening, if they were known active smokers or smoked in the past 6-months, had signs and symptoms of a respiratory infection, anemic (hemoglobin<8g/dl), were suspected or known to have chronic obstructive pulmonary disease (COPD), were suspected, or known to be pregnant, and had contraindications for bronchoscopy. All participants provided written informed consent and followed by clinical assessment before bronchoscopy. Due to the test and treat strategy, we were unable to recruit ART-naïve HIV-infected adults. Ethical approval was obtained from the College of Medicine Research Ethics Committee in Malawi (Protocol *P.01/18/2335*) and the Liverpool School of Tropical Medicine in the UK (Protocol *18-007*).

### Nasopharyngeal swabs collection and processing

Nasopharyngeal swab (FLOQSwabs™, Copan Diagnostics, USA) were collected and placed in 1 ml skim milk-tryptone-glucose-glycerol (STGG) media and processed as previously described (21).

### Bronchoalveolar lavage collection and cell isolation

BAL was collected and processed as previously described (22). Briefly, topical lignocaine spray was applied to the nasal and pharyngeal mucosa of semi-recumbent participants. A fibre-optic bronchoscope (Olympus, UK) was passed down trachea and lignocaine applied to the vocal cords and larger airways. The bronchoscope was passed down to the level of sub-segmented bronchus of the right middle lobe and four 50ml aliquots of sterile normal saline at 37°C was instilled and lavage fluid removed using gentle hand suction. BAL fluid was immediately transported to the lab on ice.

Briefly, BAL was sterile filtered and centrifuged at 500*g* at 4°C. After centrifugation, supernatant was removed, and whole cell pellet resuspended in cold PBS and centrifuged at 500*g* at 4°C. Following washing, the cell pellet was further resuspended in RPMI (Sigma-Aldrich, UK) supplemented with 10% heat inactivated fetal bovine serum and antibiotics (amphotericin, penicillin, and streptomycin) (Sigma-Aldrich, UK). Cell count, primarily of viable alveolar macrophages (based on morphology and trypan blue staining) was performed using the KOVA^®^ disposable counting chamber under bright field microscope (Olympus, Japan). Harvested airway cells, primarily AMs (23) were seeded in 12-well cell culture plates (1x10^6^/well) containing RPMI supplemented with 10% heat inactivated fetal bovine serum without antibiotics (henceforth referred to as infection media) and incubated at 37°C in 5% CO_2_ for 3 hours to allow AM adherence prior to infection studies. Due to limitation of cells retained in some individuals, not all experiments were done on all samples.

### Bacteria opsonization

For IgG opsonization, 1x10^8^ colony forming units (cfu) ST3 were opsonized with 25% of 007SP human anti-pneumococcal capsule reference serum (NIBSC, UK) and 10% intravenous immunoglobulin (IVIG) (NIBSC, UK) in infection medium as previously reported (12, 24, 25). The ST3, 007SP and IVIG suspension was co-incubated for 30 minutes in a horizontal shaking incubator (ThermoFisher Scientific, USA) at a temperature of 37°C and a speed of 170rpm.

### Fluorescent staining of pneumococci

The bacteria pellet (1x10^8^ cfu) in an aluminum foil wrapped 15 ml falcon tube was fully resuspended in 10μM CellTracker™ Red CMTPX (Invitrogen, USA) and incubated at 37°C for 1 hour, whilst gently shaking at 170rpm to ensure even staining of bacteria. Once stained, the bacteria were centrifuged twice at 4,000*g* to concentrate the stained bacterial pellet. The pellet was further resuspended in Hanks balanced salt solution (Sigma-Aldrich, UK) supplemented with 0.2% bovine albumin serum (Sigma-Aldrich, UK) and washed twice at 4,000*g*. After the final wash, stained ST3 was resuspended in 100μl infection media.

### Bacterial extracellular killing assay

To determine the growth inhibition of extracellular ST3 by airway cells, rested airway cells were infected with opsonized ST3 at MOI 50 bacteria per AM in 1ml of infection medium. Thereafter, 12-well culture plates were centrifuged for 3 minutes at 561*g* to initiate intimate contact between the phagocytes and the ST3. Plates were incubated at 37°C in 5% CO_2_ for up to 24 hours with gentle horizontal shaking 50rpm. After each hour and for three continuous hours post infection (p.i), supernatants matching the corresponding hour was removed, and wells washed three times with PBS to remove non-adherent bacteria. Supernatant were centrifuged, discarded and viable outgrowth ST3 quantified using quantitative culture to determine number of cfu (26, 27). This was done for time points 1, 2, 3 and 24 hours p.i.

### *Ex vivo* gentamicin protection assay

To determine the number of viable intracellular pneumococcal ST3 within AM, rested adherent AM were infected with opsonized ST3 at MOI 50 in 1ml of infection media. Thereafter, the plates were centrifuged at 561*g* to initiate an immediate contact between the phagocytes and the ST3. Plates were incubated at 37°C in 5% CO_2_ with gentle horizontal shaking 50rpm. One hour p.i, the supernatant and non-adherent cells were removed by aspiration, washing three times with fresh PBS. Infection media was added to the wells with adherent cells followed by 100µg/ml of gentamicin, a dose shown to kill extracellular pneumococci in this assay (27, 28), including ST3 (29–31). Plates were further incubated for 30 minutes at 37°C in 5% CO_2_ with gentle horizontal shaking 50rpm. Thirty minutes post gentamicin exposure, the wells were washed twice with sterile PBS to remove any traces of gentamicin and viable extracellular bacteria, leaving phagocytes with internalized ST3. To the adherent cells in the wells of the cell culture plates, fresh infection media with no antibiotics was added and incubation continued for 24 hours. At times 1, 2, 3 and 24 hours p.i, adherent cells were lysed with 2% saponin solution followed by quantitative culture to determine number of viable intracellular ST3 quantified.

### Flow cytometry

Flow cytometry-based immunophenotyping was used to characterize cells associated CMTPX-stained pneumococci. Briefly, cells co-incubated with CMTPX-stained ST3 were harvested and adjusted to 1x10^6^/50μl. They were then stained with a cocktail of the following antibodies: anti-human CD206 FITC (Clone 15-2, Cat no. 321104), anti-human CD163 BV421 (Clone GHI/61, Cat no. 333612), anti-CD66b APC (Clone G10F5, Cat no. 305118) (All BioLegend, UK). Cells were incubated in the dark thereafter resuspended in cold PBS and spun in the centrifuge at 500*g*. Supernatant was gently poured off and cells were gently resuspended and fixed in 0.5% paraformaldehyde and acquired on a BD Fortessa flow cytometer (Beckman Dickinson, USA).

### Confocal microscopy

Fixed airway slides were incubated with 0.5% wheat germ agglutinin (WGA; Alexa Flour^®^ 633, Thermo scientific) to stain sialic acid and N-acetylglucosaminyl residues on the cell membranes. Following incubation and washing, slides were permeabilized with 0.1% Triton X-100. Slides were subsequently washed three times with PBS before incubating the cells for 1 hour with pneumococcal omnisera/serotype 3 specific serum (SSI Diagnostica, Denmark) diluted in 5% goat serum (blocking solution). Afterwards, the slides were washed with PBS before staining for 45 minutes with a secondary monoclonal antibody conjugated with fluorochrome (anti-rabbit Alexa Flour^®^ A488, Thermo Scientific) to detect bacteria. For slides requiring AMs to be identified, anti-human CD206 (Alexa Flour^®^ 568; Thermo Scientific) was co-incubated with bacterial secondary conjugated antibody. Slides were then washed three times with PBS and once in distilled water and mounted with media containing 4′,6-diamidino-2-phenylindole (DAPI; Thermo Scientific ProLong™ Gold Antifade Mountant) and a coverslip added. Nail polish was used to seal the slide before being acquired on the Olympus FluoView 1000 confocal laser scanning fluorescent scanning microscope using a x40 objective. Z-stack images using Huygens Essential deconvolution software version16 (Scientific Volume Imaging) and viewed in Imaris 3D reconstruction software 9.4 (Bitplane).

### Statistical analysis

Data visualization and statistical analyses were performed in GraphPad Prism software (version 9.4). Descriptive statistics were used to for continuous variables by calculating medians and interquartile ranges. Groups were compared using non-parametric tests (Wilcoxon rank sum or Wilcoxon signed-rank test, Kruskal Wallis tests) depending on the distribution. For multiple pairwise comparisons, the Dunn test was used. Categorical data were summarized as proportions (and 95% confidence intervals were appropriate) and compared using the χ2 tests. Correlations were assessed using Spearman test. Effects were considered statistically significant when the *p* value was less than 0.05.

## Results

### Participant demographics and characteristics

As shown in **Table 1**, a total of 76 asymptomatic individuals were recruited, 31 HIV-uninfected, 29 PLHIV on short-term antiretroviral therapy (ART) (<3 months) and 16 PLHIV on long-term ART (>3 years). The median duration of ART in PLHIV on short-term treatment was 29.2 days (IQR, 7.75 – 48.75 days), while those on long-term treatment was 7.5 years (IQR, 4.25 – 10.75 years). Plasma HIV viral load was undetectable in 22/45 (49%) of the PLHIV on ART, with 75% (12/16) of individuals on long-term ART having undetectable viral load.

**Table 1:** Demographic and laboratory characteristics of the population.

Characteristic	HIV-uninfected (n=31)	PLHIV <3m ART (n=29)	PLHIV >3yrs ART (n=16)	*p* value
Age median (IQR)	33.0 (22 – 41)	31.5 (27.0 – 38.3)	41.0 (38.5 – 46.5)	**0.021**
Gender (Female) n (%)	11 (35.9)	16 (53.5)	11 (68.8)	**0.0001** ^*^
CD4+ count median (IQR)	640.0 (515.0 – 829.0)	488.0 (335.0 – 564.0)	619 (399.5 – 724.0)	**0.0015**
Undetectable Plasma HIV viral RNA (%)^#^	n/a	10 (37.0)	12 (75.0)	**0.0268** ^*^
BAL volume median (IQR)	122.0 (108.5 – 135.0)	130.0 (115.0 -140.0)	120.0 (105.0 -135.0)	0.3519

*Analyses were done using χ^2^- test including gender and viral load and the rest of the analysis was done using Kruskal-Wallis.

ART, antiretroviral therapy; BAL, bronchoalveolar lavage; CD, cluster of differentiation; IQR, interquartile range; PLHIV, People living with HIV. ^#^Limit of plasma viral load (log_10_) <2.42 copies/ml. The bold values represent statistical significance i.e. the p values are less than 0.05.

### Airway cells differentially control pneumococcal outgrowth during early and late infection phases

**Figure 1:**
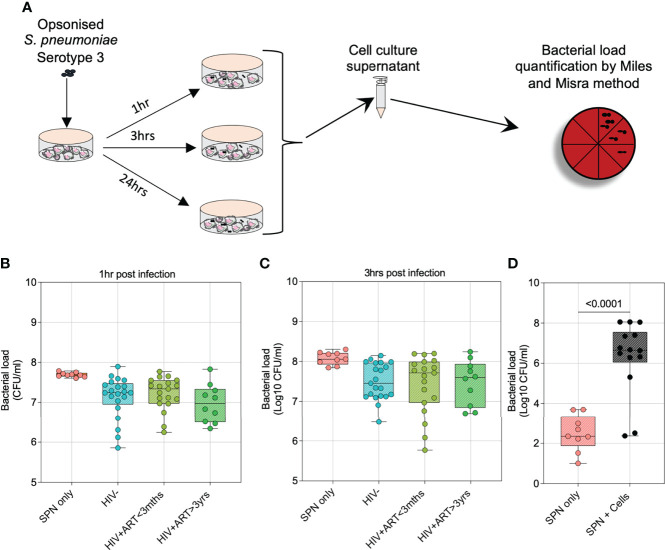
Airway cells suppress pneumococcal outgrowth better during early phases of infection. To compare the growth kinetics of *S*. *pneumoniae* Serotype 3 in the presence and absence of human airway cells, bacteria were grown in a co-culture experiment and viable counts determined at the time points indicated. Prior to incubation and co-culture experiment, bacteria were IgG-opsonized. **(A)** Schematic outlines the experimental design for pneumococcal growth kinetics in the presence and absence of human airway cells. **(B)** Airway cells suppress pneumococcal growth 1-hour post infection (p.i). **(C)** Airway cells suppress pneumococcal growth 3 hours p.i. **(D)** Differential airway cell control of *S*. *pneumoniae* 24 hours p.i, bacterial detection limit was 33 cfu/ml. In a subset of individuals with detectable bacteria, pneumococcal bacterial burden was compared in conditions grown with or without airway cells. Boxplots represent the median (centre line) and interquartile range (box), minima and maxima (whiskers). Asymptomatic HIV-uninfected (n=21); asymptomatic HIV-infected on ART<3 months (n=19); asymptomatic HIV-infected on ART>3 years (n=10); control bacteria (1 hour and 3 hours p.i, n=9; 24 hours p.i, n=25). Statistics were calculated using Kruskal-Wallis, Dunn multiple comparison test and Mann-Whitney test.

To examine the kinetics of airway cellular control of pneumococcal outgrowth, we used an invasive pneumococcal serotype 3 (ST3) isolate and lower airway cells obtained *via* bronchoalveolar lavage (BAL). Pneumococcal ST3 was grown in the presence and absence of human airway cells and bacterial load was measured in the supernatant at 1, 3 and 24 hours p.i (**Figure 1A**). In the first 3 hours p.i (early infection phase), the bacterial burden was significantly lower in conditions where pneumococci were co-cultured with human airway cells than from those without airway cells (all *p<0.007*) (**Figures 1B, C**; **Supplemental Figure 1**). Moreover, no significant differences were observed in the early control of pneumococcal outgrowth by cells obtained from HIV-uninfected adults compared to PLHIV (all *p>0.05*) (**Figures 1B, C**). However, at the 24-hour time point (late infection phase), in conditions with detectable bacteria in the supernatant, the bacterial load was significantly higher in those co-cultured with airway cells (irrespective of HIV status) than those without cells (*p<0.0001*) (**Figure 1D**). This data indicated that airway cells suppress pneumococcal outgrowth during the early phase of infection but could lose control in the late infection phase.

### AMs are the principal phagocyte associated with pneumococci during early infection phase

**Figure 2:**
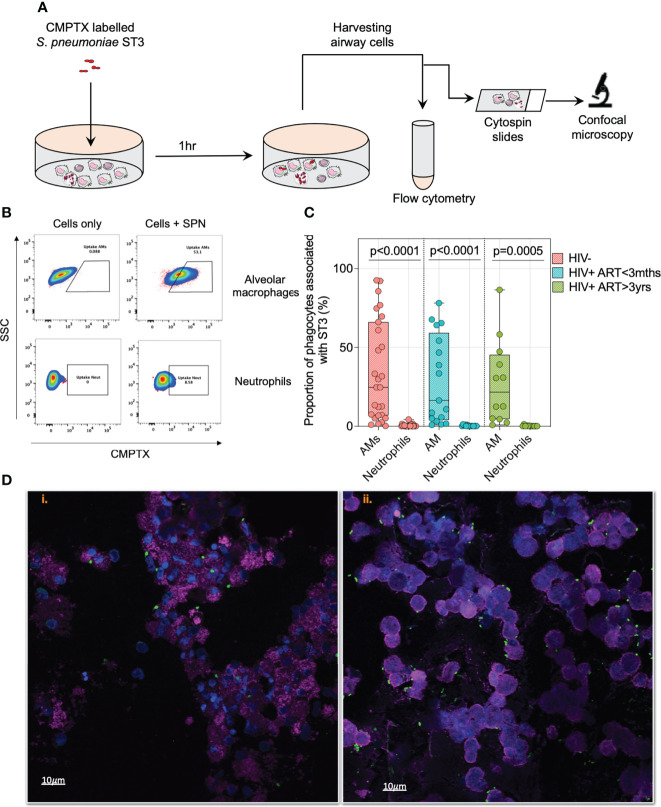
Alveolar macrophages are the predominant airway phagocyte associated with early control of pneumococci. **(A)** Schematic outlines experimental design. Briefly, IgG opsonised CMTPX stained *S. pneumoniae* were co-cultured with human airway cells for 1 hour followed by flow cytometry and confocal microscopy analysis. Prior to the infection studies, *S*. *pneumoniae* was stained with red CMPTX, followed by IgG opsonization. **(B)** Representative flow cytometry plots showing pneumococcal uptake by CD206+CD163+ AMs and CD66b+ neutrophils at MOI 50. Proportion of AMs/neutrophils associated with pneumococci were identified by subtracting the gating frequency of uninfected AMs/neutrophil from cells infected with CMTPX stained ST3. **(C)** Paired comparison for the frequency of AMs and neutrophils associated with pneumococci at MOI 50 post *ex vivo* infection. **(D)** Representative single fluorescent field images taken from a 1 individual showing the association of alveolar cells (WGA; cell membranes – purple; DAPI – nucleus) with pneumococci-ST3 (bacteria – green) at i) MOI 10 and ii) MOI 50 (at magnification x63). Scale bars: left panel =10μm; right panel =10μm. Cells associated with bacteria are shown with the arrow (orange). Boxplots represent the median (centre line) and interquartile range (box), minima and maxima (whiskers). Asymptomatic individuals; HIV-uninfected (n=25), HIV-infected on ART <3 months (n=15), HIV-infected ART>3 years (n=12). Statistics were calculated using Wilcoxon signed rank test.

To identify the airway immune cells associated with early control of pneumococcal growth, we stained pneumococcal ST3 with CMTPX dye and co-incubated them with airway cells for 1 hour to identify the predominant immune cells that bound or internalized pneumococci (**Figure 2A** and **Supplemental Figure 2**). Irrespective of HIV status, AMs were the principal phagocyte associated with IgG-opsonized ST3 as observed by flow cytometry (**Figures 2B, C**). This was also visualized using confocal microscopy at 1 hour post incubation, showing binding and internalization of fluorescent IgG-opsonized ST3 to cells morphologically analogous to AMs (**Figure 2D** and **Supplemental Video 1**). These results demonstrated that AMs were the predominant airway phagocyte associated with early control of pneumococcal outgrowth.

### Pneumococcal ST3 intracellular survival in human AMs following *ex vivo* infection

**Figure 3:**
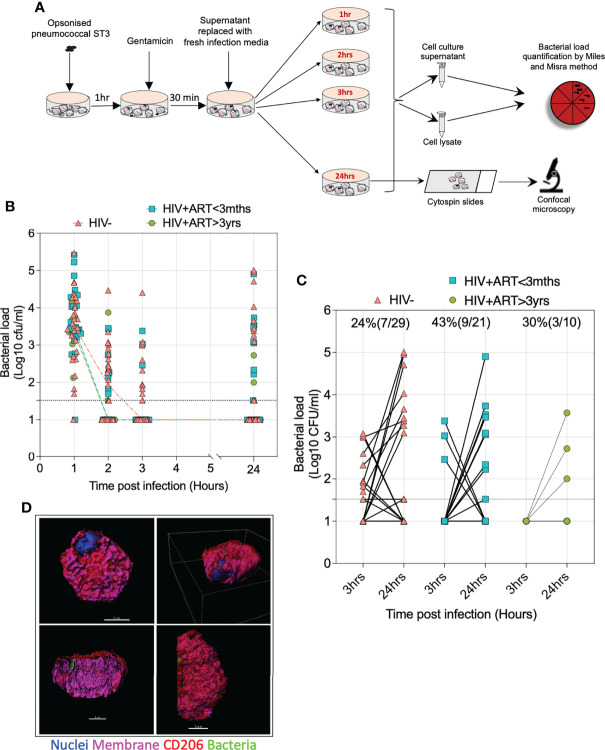
Intracellular survival of *S. pneumoniae* within AMs. **(A)** Schematic outlines the gentamicin protection experimental design. **(B)** Graph depicts the AMs intracellular bacterial load p.i at the time points indicated. **(C)** Samples of AMs from individuals with undetectable bacteria at 3 hours post infection (p.i) but having detectable pneumococcal persistence at 24 hours p.i. **(D)** Representative 3D Z-stack images on different planes demonstrating internalised *S. pneumoniae*-ST3 within CD206^+^ AMs (WGA; cell membranes – purple; DAPI – nucleus, red – monoclonal anti-CD206) with *S. pneumoniae*-ST3 (bacteria capsule, FITC– green). Scale bars, top left panel =5μm; top right panel =3μm, bottom left panel =3μm; bottom right panel =3μm. Asymptomatic individuals; HIV-uninfected (n=29), HIV-infected on ART <3 months (n=21), HIV-infected ART>3 years (n=10). Statistics were calculated using Chi-squared test and Wilcoxon signed rank test.

Next, we sought to assess intracellular survival of pneumococci in AMs from PLHIV and HIV-uninfected adults using the gentamicin protection assay (GPA). In this assay, IgG-opsonized viable ST3 at MOI 50 were incubated with AM for 1 hour, gentamicin was added for 30 minutes, then the cell culture supernatant was removed, replaced with gentamicin-free cell culture media and cells incubated for a further 1, 2, and 24 hours (**Figure 3A**). Following lysis of the AM and bacterial culture at the different time points, we observed a reduction in the intracellular bacterial load from 1 to 3 hours p.i, irrespective of HIV status (HIV- 3100cfu/ml vs. 10cfu/ml; HIV+ART+<3m 3039cfu/ml vs. 10cfu/ml; HIV+ART+>3yr 2228cfu/ml vs. 10cfu/ml) (**Figure 3B**). In a subset of individuals, we observed that bacteria were undetectable at 3 hours p.i within the AMs but detectable at 24 hours p.i (HIV- 24% vs. HIV+ART+<3m 43% vs. HIV+ART+>3yr 30%) (**Figure 3C**). We ascertained presence of intracellular pneumococci in AMs 24 hours p.i using confocal microscopy, showing that pneumococci were present intracellularly within CD206^+^ airway cells (**Figure 3D**). CD206 is a classic marker of AMs (32–35).

### *In vivo* evidence of intracellular survival of pneumococci in human AMs

**Figure 4:**
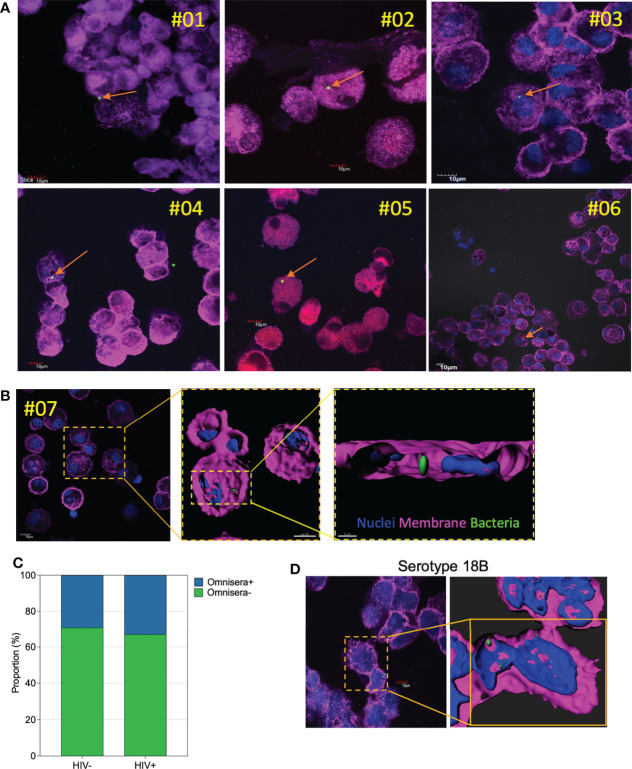
*In vivo* survival and detection of pneumococci within AMs. Representative single confocal microscopy images taken from a subset of individuals showing presence of pneumococci within cells analogous to AMs. **(A)** A subset of 6 individuals showing *in vivo* internalized pneumococci (arrow) within AMs. **(B)** Representative single confocal microscopy image showing internalized pneumococci (dashed lines) within AMs. Three-dimensional reconstruction through deconvolution analysis shows the localization of the pneumococci within the AMs from the upper focal plane. The same three-dimensional reconstruction clarifies the localization of the pneumococci within the AMs. **(C)** Proportion of AMs testing positive for pneumococcal omnisera (HIV-uninfected, n=18; treated HIV+, n=31). **(D)** A representative confocal image showing AMs positivity for serotype 18B, also identified in the nasal swabs. The three-dimensional reconstruction image of the pneumococci serotype 18 within the AMs. WGA; cell membranes – purple; DAPI – nucleus; with pneumococci-ST3 (bacteria – green) at magnification x63. Scale bars for panel A, all 10μm; panel B, top right and left =10μm, bottom right =5μm; panel D, left panel=10μm and right panel=5μm.

To corroborate the above observations, we explored *in vivo* evidence of intracellular survival of pneumococci in AMs from otherwise asymptomatic adults. Cytospins were prepared from freshly isolated BAL samples aiming to deposit 3 x 10^4^ cells per cytospin, mostly AMs, onto the slides. We stained cytospin slides, with pan-pneumococcal antibodies (Omnisera), wheat germ agglutinin (WGA) and 4′,6-Diamidino-2-phenylindole dihydrochloride (DAPI) and found that almost one third of the samples had detectable pneumococci with counts ranging from one to six bacterial cells (HIV- 33% (6/18) vs. treated HIV+ 29% (9/31)) (**Figures 4A–C**). Microscopy analysis found that in all samples the pneumococci were associated to cells morphologically consistent with AMs (**Figures 4A, B**). For eight of the BAL samples, Z-stack analysis and 3D reconstruction were performed, which showed pneumococci were located within the AMs (**Supplemental Figure 4**, **Supplemental Video 2**). We also stained a slide from an individual carrying pneumococcal serotype 18 in their nasopharynx with serotype-specific antibodies and microscopically detected ST18 pneumococci intracellularly in matched AMs (**Figure 4D**). Collectively, these data confirm pneumococcal survival in primary human AMs.

### Pneumococcal intracellular survival in human AMs is associated with continued propagation of infection

**Figure 5:**
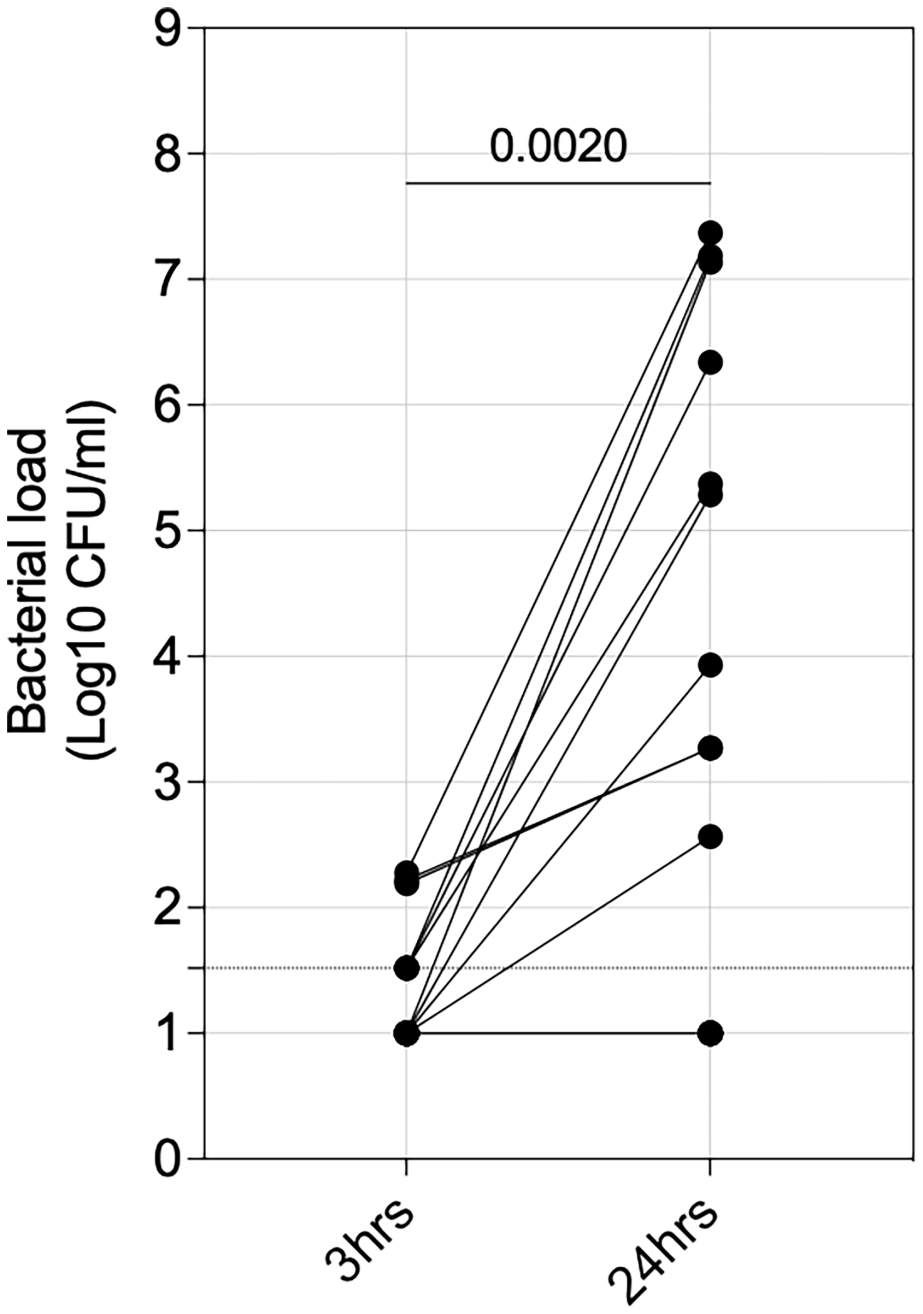
Propagation of extracellular pneumococci. Alveolar macrophages (AMs) were co-incubated with IgG-opsonised viable ST3 at MOI 50 for 1 hour, gentamicin was added for 30 minutes, then the cell culture supernatant was removed, replaced with gentamicin-free cell culture media and cells incubated. The graph shows outgrowth of pneumococci in cell culture supernatant at 3- and 24 hours (n=17). Statistics were calculated using Wilcoxon signed rank test.

We next sought to assess the impact of intracellular survival of *S. pneumoniae* on propagation of infection. We measured the presence of pneumococci in a random subset of culture supernatants (irrespective of HIV status; n=17) from the gentamycin protection assay and observed that 59% (10/17) of the individuals had an increase (*p=0.0020*) in bacterial load from 3 to 24 hours p.i (**Figure 5**), suggestive of bacterial replication. Together, these data indicated that intracellular survival of pneumococci in AMs could propagate pneumococcal infection.

### Primary human AMs exhibit diminished control of intracellular pneumococci

**Figure 6:**
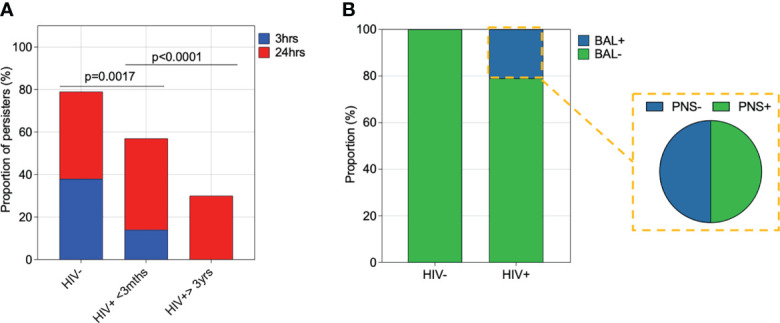
Impact of HIV infection on pneumococcal intracellular survival in alveolar macrophages. **(A)** Alveolar macrophages (AMs) were co-incubated with IgG-opsonised viable ST3 at MOI 50 for 1 hour, gentamicin was added for 30minutes, then the cell culture supernatant was removed, replaced with gentamicin-free cell culture media and cells incubated. Proportion of individuals with viable ST3 at 3 and 24 hours following lysis of AMs. HIV-uninfected (n=29), HIV-infected on ART <3 months (n=21), HIV-infected ART>3 years (n=10). Statistics were calculated using Chi-squared test. **(B)** AMs not exposed to experimental ST3 were cultured for 24 hours and subsequently lysed for the detection of viable intracellular pneumococci by microbiological culture. A higher proportion of Individuals with treated HIV had pneumococci in their AMs compared to the HIV-uninfected individuals (HIV+ n=31, HIV- n=18). Fifty percent (n=6) of the individuals with viable pneumococci in AMs also had viable bacteria on their nasal swabs.

Finally, we investigated the impact of HIV infection on survival of pneumococcal intracellular survival in human AMs. First, we calculated the proportion of individuals from whom pneumococci was still detectable at 3 and 24 hours p.i following lysis of AMs in the gentamycin protection assay. The proportion of individuals with detectable pneumococci substantially increased between 3 to 24 hours p.i in PLHIV compared to HIV-uninfected adults (*p=0.0034*) (**Figure 6A**). Second, we assessed *in vivo* bacterial survival in AMs by culturing primary human AM from asymptomatic adults for 24 hours, followed by cell lysis and subsequent microbiological culture to detect presence of any viable pneumococci from natural infection. Viable pneumococci were commonly identifiable in PLHIV than HIV-uninfected adults (HIV- 0% (0/18) vs. treated HIV+ 19% (6/31)) (**Figure 6B**), indicating *in vivo* survival of pneumococci in AM. Among the individuals with evidence of *in vivo* AM infection, 50% (3/6) had culture detectable nasopharyngeal pneumococcal carriage (**Figure 6B**). Collectively, these data suggested that AMs from PLHIV have diminished intracellular killing capacity of pneumococci.

## Discussion

The success of the pneumococcus as a pathogen depends on its commensal relationship with its obligate human host, and much of our knowledge on its commensalism is confined to the upper respiratory tract (36), with minimal data from the lower respiratory tract in humans. Data from the experimental human challenge model have shown that pneumococci can survive *in vivo* in human AMs, without causing overt disease (12). This raises the question of whether AMs are a potential reservoir of pneumococcal persistence in the human airways, and whether HIV infection promotes persistence of this intracellular reservoir leading to increased risk of pneumococcal pneumonia. Our study sheds new light on the intracellular survival of *S. pneumoniae* in human AMs from a high pneumococcal transmission and disease-burdened African setting. Using both *ex vivo* and *in vivo* approaches, our study demonstrates that pneumococci survive intracellularly in human AMs, and that this is more frequent in AMs from PLHIV than HIV-uninfected adults.

The mechanisms for pneumococcal intracellular survival in macrophages are still not fully understood, despite a growing recognition of the ability of pneumococci to survive intracellularly in macrophages (12, 14). However, there is some evidence suggesting that pneumolysin, a cholesterol binding and pore-forming toxin, facilitates intracellular lysosomal escape of the bacteria in macrophages and dendritic cells (37, 38). Specifically, pneumolysin binds to the mannose receptor (CD206), widely found on AMs, thus facilitating uptake of pneumococci, but also dampening the inflammatory cytokine responses through upregulation of cytokine suppressor-1 (SOCS1) that leads to attenuated intracellular bacterial killing (38). It has also been shown that *S*. *pneumoniae* can attenuate host autophagic degradation of intracellular pneumococci for its survival within cells (39). Moreover, the non-hemolytic pneumolysin strains of *S*. *pneumoniae* survive better inside both alveolar epithelial cells and THP-1 macrophages than the hemolytic pneumolysin strains (37). Thus, these findings suggest that this prototypical extracellular bacterium could have an intracellular niche within macrophages and potential other cells for its propagation and maintenance *in vivo.* As shown in this study and in the experimental human challenge model that this intracellular niche is also present in healthy adults (12), this could indicate that in the presence of an intact immune system, bacterial persistence does not always translate into disease; akin to latent tuberculosis.

On the other hand, PLHIV are at an increased risk of pneumococcal pneumonia and exhibit almost 2-fold higher pneumococcal carriage prevalence than HIV-uninfected adults (1, 5, 7, 40, 41). We have shown that intracellular persistence of *S. pneumoniae* within AMs *ex vivo* and *in vivo* is more frequent in PLHIV than HIV-uninfected adults. This is consistent with observations demonstrating HIV-associated impairment of bactericidal killing mechanisms of macrophages (42–45). Myeloid cell leukemia 1 (mcl-1), an anti-apoptotic protein, is upregulated in PLHIV, and has been shown to interfere with the late bactericidal killing mechanism of AMs (42–44). While, HIV gp120 inhibits mitochondrial-ROS (mROS) dependent intracellular killing of *S. pneumoniae* in monocyte-derived macrophages (MDM) (43). Considering that HIV persists in the airway even following suppressive ART (45), the diminished intracellular killing of pneumococci in AMs in PLHIV on ART could be due to the presence of HIV or its viral components in the airway. It is therefore plausible that intracellular persistence of pneumococci in AMs could contribute to the increased risk of pneumococcal pneumonia in PLHIV due to its potential to propagate pneumococcal infection.

A major strength of this work is the use of a locally-relevant and globally-important invasive pneumococcal ST3. ST3 is among the commonest pneumococcal carriage serotypes in Malawi (7) and is also a global concern due to the persistent ST3 disease (7, 10, 11). Pneumococcal ST3 is biochemically different from the other serotypes, having a large thick polysaccharide capsule, surrounded by a slime layer, which gives it a characteristic mucoid appearance when grown on blood agar plates (46, 47). The polysaccharide capsule is a major virulence factor of the pneumococci as it inhibits complement and antibody mediated opsonophagocytosis (48–51). Despite Pneumococcal ST3 being biochemically different, our findings are consistent with observations from other serotypes including serotype 1 (37), serotype 2 (43, 44), serotype 6B (12) and serotype 18, that also survive intracellularly, suggesting intracellular survival may not be capsular specific but is common amongst pneumococcal serotypes.

A potential limitation to the study is the use of unprotected bronchoscopes, which raises a possibility of BAL contamination with components from the upper respiratory tract. As a result, identification of intracellular pneumococci in AM from natural infection could be a sampling artefact, but we think this is highly unlikely. Notably, we did not observe overgrowth of contaminants including bacteria or fungi in our BAL cell culture 24 hours p.i.

In conclusion, our study shows intracellular survival of *S. pneumoniae* in primary human AMs is common, but it is exacerbated in PLHIV. The findings suggest that diminished intracellular killing of pneumococci in AMs could in part contribute to the increased propensity of pneumococcal pneumonia in PLHIV. Our findings have implications on our understanding of pneumococcal pathogenesis for this prototypical extracellular organism and indicate that development of preventative therapeutic approaches should consider the existence of intracellular niches for the pneumococcus.

## Data availability statement

The original contributions presented in the study are included in the article/**Supplementary Materials**. Further inquiries can be directed to the corresponding authors.

## Ethics statement

The studies involving human participants were reviewed and approved by the College of Medicine Research Ethics Committee in Malawi (Protocol P.01/18/2335) and the Liverpool School of Tropical Medicine in the UK (Protocol 18-007). The patients/participants provided their written informed consent to participate in this study.

## Author contributions

KJ, TiN, HM, MO, ToN, MO and DF designed the study. KJ and MO supervised the work. TiN, LS, JP, MC, ZJ and CM processed all the samples and performed experiments. TiN, MC, ZJ, MO and KJ carried out the data management and statistical analysis. TiN and KJ wrote the initial manuscript draft. All authors contributed to the article and approved the submitted version.

## Funding

KJ was supported by the Wellcome (UK) through an Intermediate Fellowship 105831/Z/14/Z. TiN was supported by a training grant awarded as part of the Wellcome Strategic award to Malawi-Liverpool-Wellcome Trust Clinical Research Programme 101113/Z/13/Z084 and the Legacy Award from the Federation of African Immunological Societies. HM was supported by the Medical Research Council (MRC, UK) through an African Research Leader award MR/PO20526/1. MC and MO were supported by an IDRF grant. MC was supported by a HIC-Vac training grant PS3187 and funds from the University of Leicester Doctoral College.

## Acknowledgments

The authors would like to thank all study volunteers, the clinical team and supporting staff of Malawi-Liverpool-Wellcome Trust Clinical Research Programme and Queen Elizabeth Central Hospital for their support and cooperation during the study.

## Conflict of interest

The authors declare that the research was conducted in the absence of any commercial or financial relationships that could be construed as a potential conflict of interest.

## Publisher’s note

All claims expressed in this article are solely those of the authors and do not necessarily represent those of their affiliated organizations, or those of the publisher, the editors and the reviewers. Any product that may be evaluated in this article, or claim that may be made by its manufacturer, is not guaranteed or endorsed by the publisher.

## Supplementary material

Supplementary Figure S1
*S. pneumoniae* outgrowth in the presence and absence airway cells. Growth curves were performed to measure growth of *S. pneumoniae* in the presence and absence of airway cells. Asymptomatic HIV-uninfected (n = 21); asymptomatic HIV-infected on ART<3 months (n = 19); asymptomatic HIV-infected on ART>3 years (n = 10); control bacteria (n = 9).

Supplementary Figure S2 Gating strategy for AMs and neutrophils Identification of CD206+CD163+ AMs and CD206-CD163-CD66b+ neutrophils in airway lumen. Human airway cells from were stained with fluorochrome-conjugated antibodies against surface markers of interest.

Supplementary Figure S3 2D and 3D images of *in vivo* internalized pneumococci within AM cell. i) Representative single confocal microscopy images taken from a 1 individual showing presence of pneumococci within cells analogous to AMs. ii) 3D single confocal microscopy images showing presence of pneumococci within cells analogous to AMs. iii) Video outlines a three-dimensional reconstruction of deconvolved Z-stack confocal images of pneumococci internalised within AMs. The cytospin samples were stained with wheat germ agglutinin (WGA; cell membranes – purple), DAPI (nucleus; blue) and FITC (green; pneumococcus capsule).

Supplementary Table 2 3D images of *in vivo* internalized pneumococci within AM cell. Representative 3D single confocal microscopy images taken from five individuals showing presence of pneumococci within cells analogous to AMs. The cytospin samples were stained with wheat germ agglutinin (WGA; cell membranes – purple), DAPI (nucleus; blue) and FITC (green; pneumococcus capsule).

Supplementary Video S1 3D video image of pneumococci binding and internalized within AMs infected *ex vivo*. Video outlines a three-dimensional reconstruction of deconvolved Z-stack confocal images of pneumococci binding and internalized within AMs. The cytospin samples were stained with wheat germ agglutinin (WGA; cell membranes – purple), DAPI (nucleus; blue) and FITC (green; pneumococcus capsule).

Supplementary Video S2 3D video image of pneumococci internalized within AMs infected *in vivo*. Video outlines a three-dimensional reconstruction of deconvolved Z-stack confocal images of pneumococci binding and internalized within AMs. The cytospin samples were stained with wheat germ agglutinin (WGA; cell membranes – purple), DAPI (nucleus; blue) and FITC (green; pneumococcus capsule).
