# Supplementary figures and images for: Intracellular survival of Streptococcus pneumoniae in human alveolar macrophages is augmented with HIV infection
Source: Front Immunol. 2022 Sep 20;13:992659. doi: 10.3389/fimmu.2022.992659 (PMC9531125; doi:10.3389/fimmu.2022.992659)

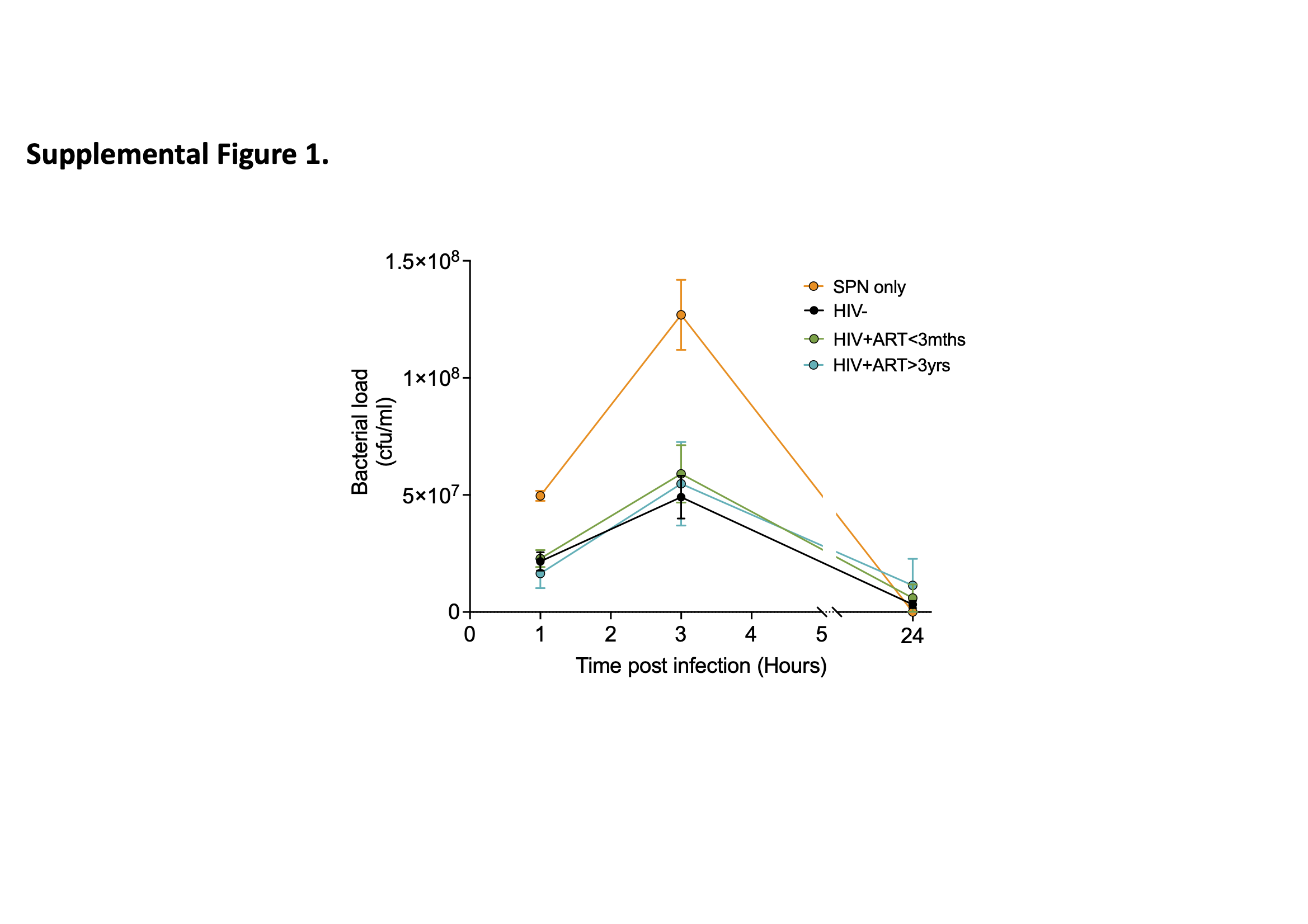

Supplement: Supplementary Figure S1 — S. pneumoniae outgrowth in the presence and absence airway cells. Growth curves were performed to measure growth of S. pneumoniae in the presence and absence of airway cells. Asymptomatic HIV-uninfected (n = 21); asymptomatic HIV-infected on ART<3 months (n = 19); asymptomatic HIV-infected on ART>3 years (n = 10); control bacteria (n = 9). [file Image_1.tiff]

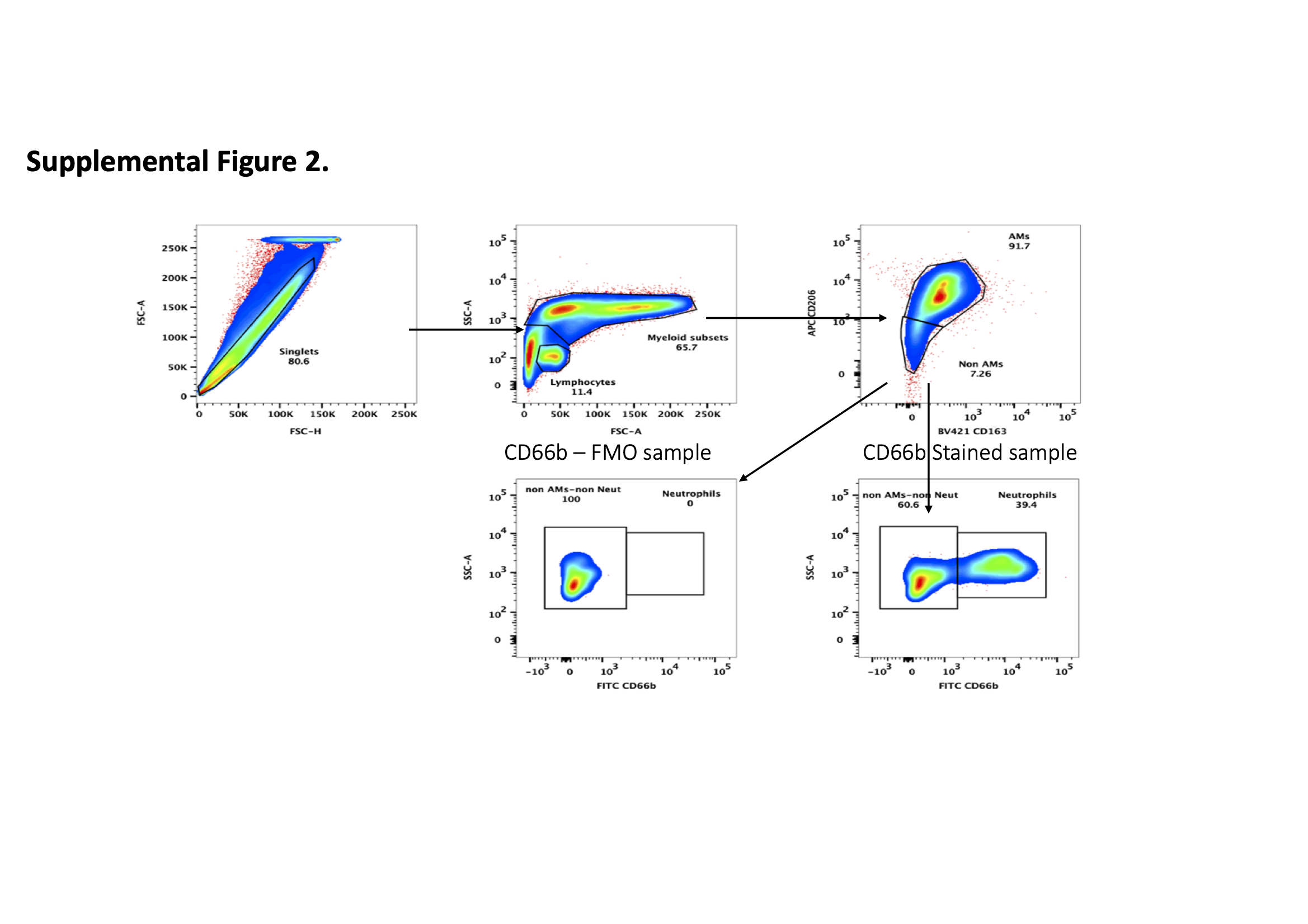

Supplement: Supplementary Figure S2 — Gating strategy for AMs and neutrophils Identification of CD206+CD163+ AMs and CD206-CD163-CD66b+ neutrophils in airway lumen. Human airway cells from were stained with fluorochrome-conjugated antibodies against surface markers of interest. [file Image_2.tiff]

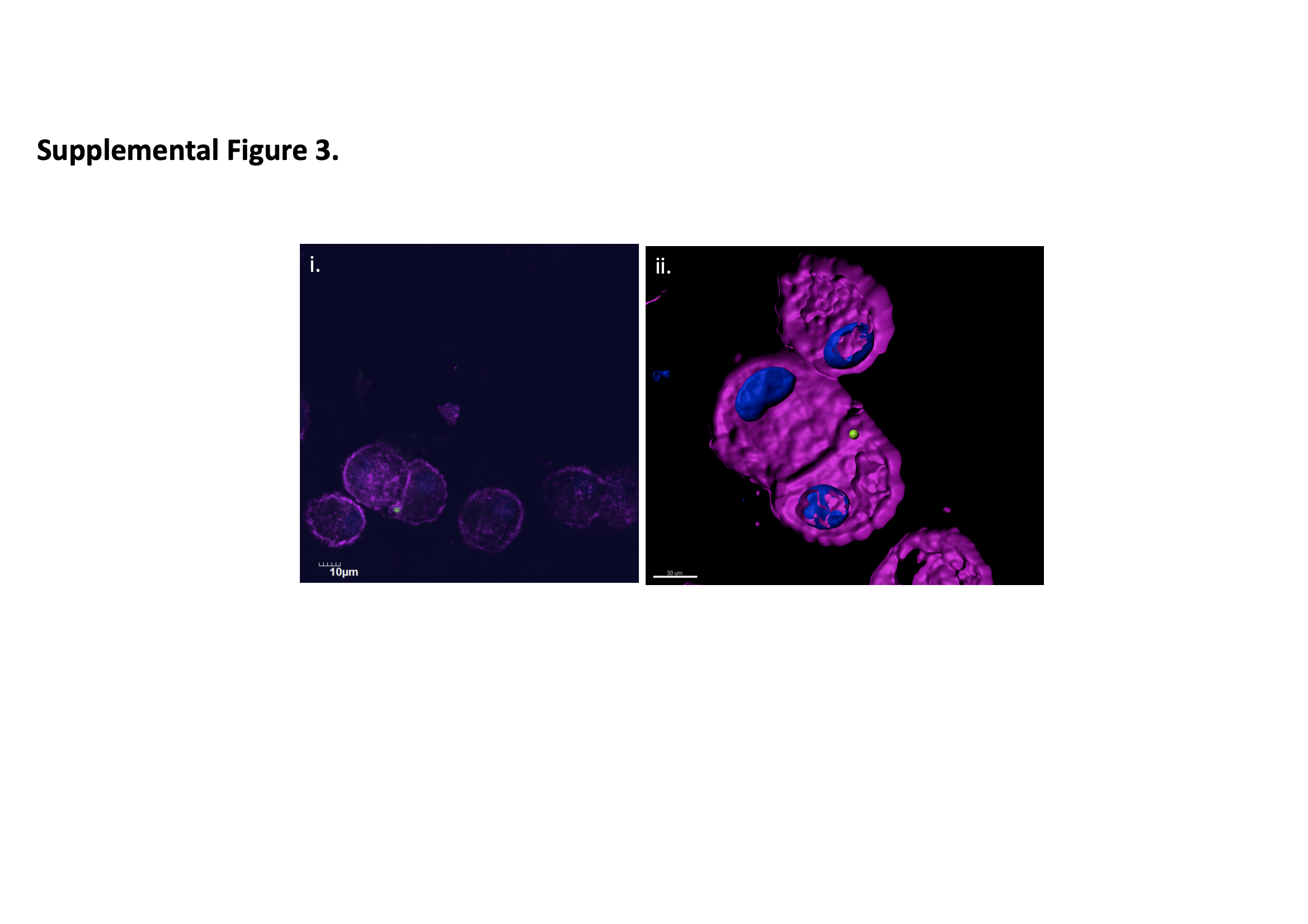

Supplement: Supplementary Figure S3 — 2D and 3D images of in vivo internalized pneumococci within AM cell. i) Representative single confocal microscopy images taken from a 1 individual showing presence of pneumococci within cells analogous to AMs. ii) 3D single confocal microscopy images showing presence of pneumococci within cells analogous to AMs. iii) Video outlines a three-dimensional reconstruction of deconvolved Z-stack confocal images of pneumococci internalised within AMs. The cytospin samples were stained with wheat germ agglutinin (WGA; cell membranes – purple), DAPI (nucleus; blue) and FITC (green; pneumococcus capsule). [file Image_3.tiff]

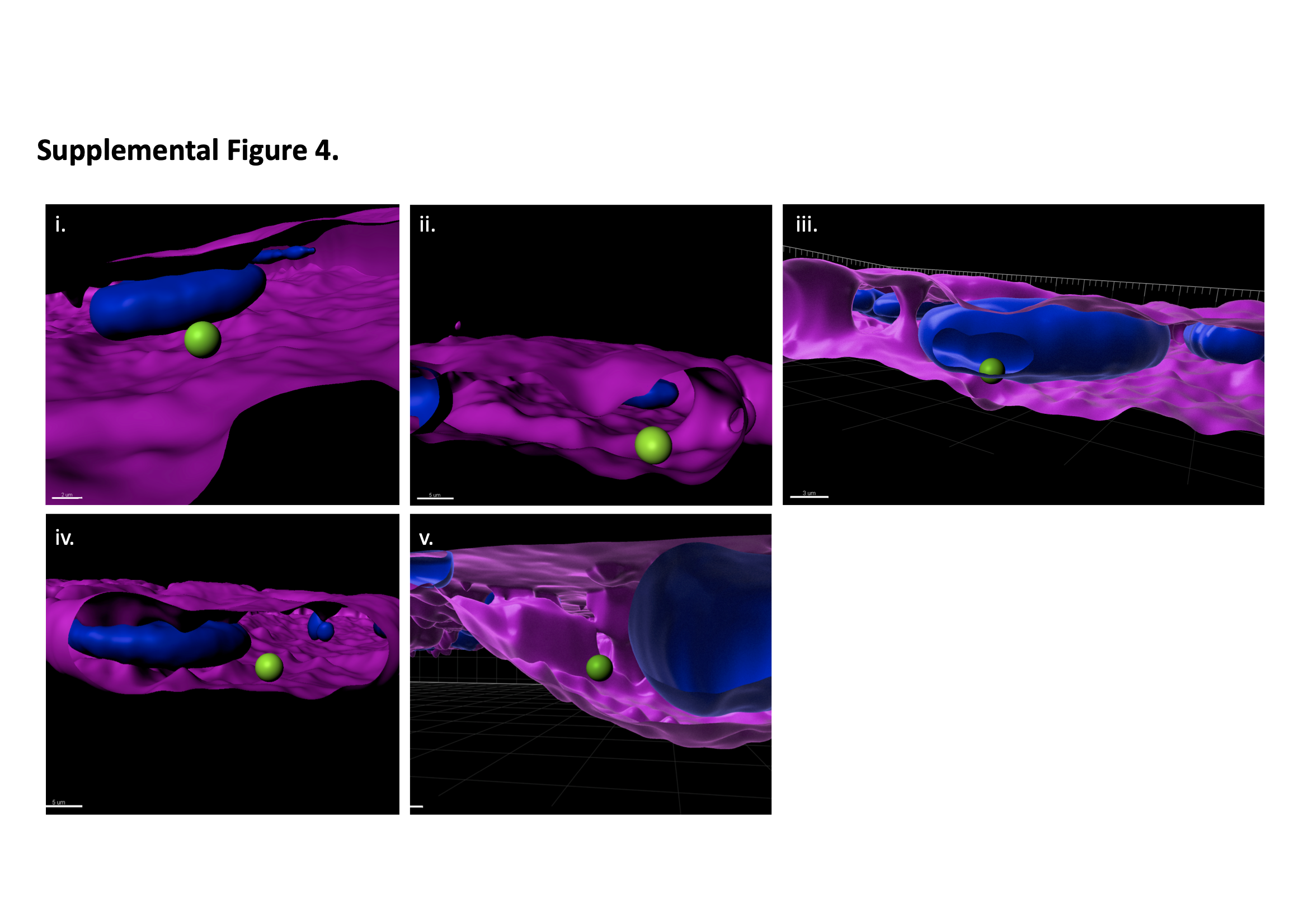

Supplement: Supplementary Table 2 — 3D images of in vivo internalized pneumococci within AM cell. Representative 3D single confocal microscopy images taken from five individuals showing presence of pneumococci within cells analogous to AMs. The cytospin samples were stained with wheat germ agglutinin (WGA; cell membranes – purple), DAPI (nucleus; blue) and FITC (green; pneumococcus capsule). [file Image_4.tiff]

## Slide 1
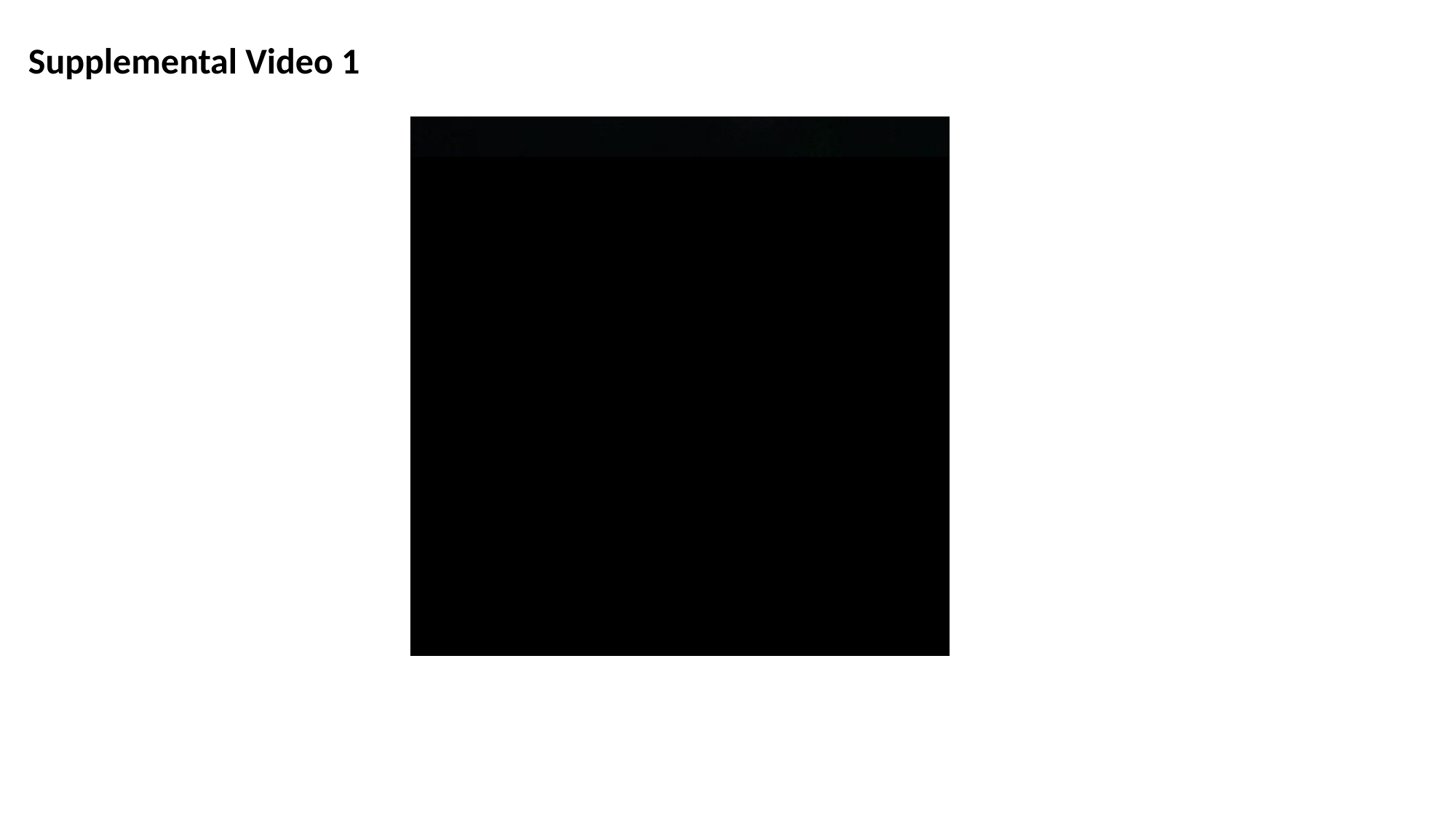

Supplemental Video 1

## Slide 2
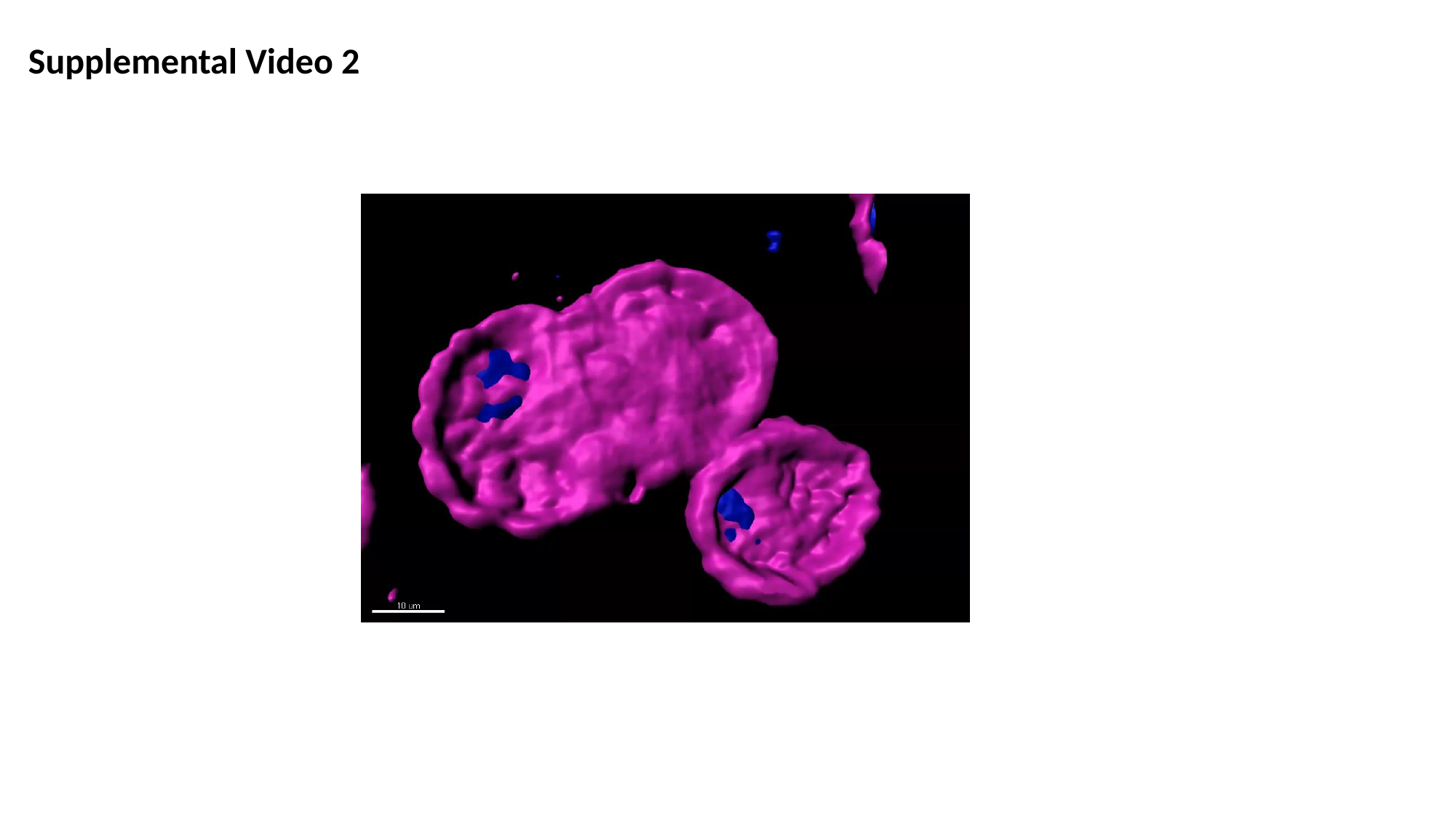

Supplemental Video 2

Supplement: Supplementary Video S1 — 3D video image of pneumococci binding and internalized within AMs infected ex vivo. Video outlines a three-dimensional reconstruction of deconvolved Z-stack confocal images of pneumococci binding and internalized within AMs. The cytospin samples were stained with wheat germ agglutinin (WGA; cell membranes – purple), DAPI (nucleus; blue) and FITC (green; pneumococcus capsule). [file Presentation_1.pptx]
